# The Efficacy of Famotidine in improvement of outcomes in Hospitalized COVID-19 Patients: A structured summary of a study protocol for a randomised controlled trial

**DOI:** 10.1186/s13063-020-04773-6

**Published:** 2020-10-13

**Authors:** Hamid Reza Samimagham, Mehdi Hassani Azad, Maryam Haddad, Mohsen Arabi, Dariush Hooshyar, Mitra KazemiJahromi

**Affiliations:** 1 Clinical Research Development Center, Shahid Mohammadi Hospital, Hormozgan University of Medical Sciences, Bandar Abbas, Iran; 2 Infectious and Tropical Diseases Research Center, Hormozgan Health Institute, Hormozgan University of Medical Sciences, Bandar Abbas, Iran; 3 Department of Internal Medicine and Public Health Research Center, Family Medicine Department, Iran University of Medical Sciences, Tehran, Iran; 4 Student Research Committee, Faculty of Medicine, Hormozgan University of Medical Sciences, Bandar Abbas, Iran; 5 Endocrinology and Metabolism Research Center, Hormozgan University of Medical Sciences, Bandar Abbas, Iran

**Keywords:** COVID-19, Randomised controlled trial, protocol, Famotidine, Hospitalized, Efficacy

## Abstract

**Objectives:**

This study aims to investigate the effect of Famotidine on the recovery process of COVID-19 patients.

**Trial design:**

This phase III randomized clinical trial was designed with two parallel arms, placebo-controlled, single-blind, and concealed allocation.

**Participants:**

All COVID-19 patients admitted to Shahid Mohammadi Hospital in Bandar Abbas whose PCR test results are positive for SARS-Cov-2 and sign the written consent of the study are included in the study and immunocompromised patients, end-stage renal disease, moderate renal failure (clearance Creatinine 30 to 50 ml/min) or stage 4 severe chronic kidney disease or need for dialysis (creatinine clearance lesser than 30 ml/min), history of liver disease, hepatitis C infection or alcoholism, Glucose 6 phosphate dehydrogenase deficiency(G6PD), the ratio of Alanine transaminase to Aspartate transaminase 5 times above the normal limit, history or evidence of long QT segment on Electrocardiogram, psoriasis or porphyria, pregnancy, use of oral contraceptives, Dasatinib, Neratinib, Ozanimod, Pazopanib, Rilpivirine, Siponimod and/or Tizanidine and allergies to any study drug are excluded.

**Intervention and comparator:**

Intervention group receives standard pharmacotherapy according to the treatment protocols of the National Committee of COVID-19 and oral famotidine 160 mg (Manufactured by Chemidarou Pharmaceutical Company) four times a day until the day of discharge, for a maximum of fourteen days. Comparator group receives standard drug therapy according to the treatment protocols of the National Committee of COVID-19 and placebo in the same dosage.

**Main outcomes:**

Patients’ temperature, respiration rate, oxygen saturation, lung infiltration, lactate dehydrogenase and complete blood count were measured at the baseline (before the intervention) and on day 14 after the intervention or on the discharge day.

**Randomisation:**

The person who has no role in admitting patients and assigning patients to random codes preparing random sequences using online tools and by permuted block randomization method. Eligibility criteria are monitored by the person responsible for admitting patients. Codes in a random sequence are assigned to patients by the treatment team without knowing that each code is in the intervention or comparator group. Patient codes are then matched to randomly generated sequence information for interventions.

**Blinding (masking):**

All participants are unaware of which group of this study they are in and after grouping patients in the groups, Patients receive Famotidine in the treatment group and receive a placebo in the control group. The lead researcher, care givers, data collectors, and outcome assessors are aware of the grouping of patients.

**Numbers to be randomised (sample size):**

As there is no prior work on this research question, so no assumptions for the sample size calculation could be made. A total of 20 patients participate in this study, which are randomly divided into two groups of 10 as intervention or control groups.

**Trial status:**

Version 3 of the protocol was approved by the Deputy of Research and Technology and the ethics committee of Hormozgan University of Medical Sciences on August 2, 2020, with the local code 990245, and the recruitment started on August 17, 2020. recruitment ended on August 31, 2020. Since the recruitment ended earlier than expected (the expected recruitment end date was 21/12/2020), we submitted post recruitment but prior to publication of the results.

**Trial registration:**

The protocol was registered before starting subject recruitment under the title: The effect of Famotidine on the improvement of patients with COVID-19, IRCT20200509047364N2, at Iranian Registry of clinical trials (https://www.irct.ir/trial/49657) on 17 August 2020.

**Full protocol:**

The full protocol is attached as an additional file, accessible from the Trials website (Additional file 1). In the interest in expediting dissemination of this material, the familiar formatting has been eliminated; this Letter serves as a summary of the key elements of the full protocol.

The study protocol has been reported in accordance with the Standard Protocol Items: Recommendations for Clinical Interventional Trials (SPIRIT) guidelines (Additional file 2).

## Supplementary information

**Additional file 1.** Full Study Protocol.

**Additional file 2.** SPIRIT 2013 Checklist: Recommended items to address in a clinical trial protocol and related documents.

## Data Availability

The authors have not yet decided on the sharing of information.

