# Supplementary material for: The Efficacy of Famotidine in improvement of outcomes in Hospitalized COVID-19 Patients: A structured summary of a study protocol for a randomised controlled trial
Source: Trials. 2020 Oct 13;21:848. doi: 10.1186/s13063-020-04773-6 (PMC7552598; doi:10.1186/s13063-020-04773-6)

**Title: The Efficacy of Famotidine in the improvement of outcomes in Hospitalized COVID-19 Patients: A structured summary of a study protocol for a randomized controlled trial**

**Authors**

Hamid Reza Samimagham^1^, Mehdi Hassani Azad ^2^, Maryam Haddad ^1^,Mohsen Arabi ^3^, Dariush Hooshyar ^4^, Mitra KazemiJahromi ^5, *^

^1^Clinical Research Development Center, Shahid Mohammadi Hospital, Hormozgan University of Medical Sciences, Bandar Abbas, Iran

^2^ Infectious and Tropical Diseases Research Center,Hormozgan Health Institute, Hormozgan University of Medical Sciences, Bandar Abbas, Iran

^3^ Department of Internal Medicine and Public Health Research Center,Family Medicine Department,Iran University of Medical Sciences,Tehran, Iran

^4^ Student Research Comitte,Faculty of Medicie, , Hormozgan University of Medical Sciences, Bandar Abbas, Iran

^5^ Endocrinology and Metablism Research Center, Hormozgan University of Medical Sciences, Bandar Abbas, Iran

*** Correspondance:**

**Mitra KazemiJahromi**

Endocrinology and Metablism Research Center, Hormozgan University of Medical Sciences, Bandar Abbas, Iran

Tel: +989177912820*

**Email:** [samimagham@yahoo.com^1^](mailto:samimagham@yahoo.com1) , [mehdihassaniazad@gmail.com^2^](mailto:mehdihassaniazad@gmail.com2) , [Dr.maryhd@gmail.com^1^](mailto:Dr.maryhd@gmail.com1) , [Arabi.m@iums.ac.ir^3^](mailto:Arabi.m@iums.ac.ir3) , [dariush.hooshyar@gmail.com^4^](mailto:dariush.hooshyar@gmail.com4) , [mitra.kazemijahromi@gmail.com^5^](mailto:mitra.kazemijahromi@gmail.com5) ^*^

**Abstract**

**Objectives**

This study aims to investigate the effect of Famotidine on the recovery process of COVID-19 patients.

**Trial design**

This phase III randomized clinical trial was designed with two parallel arms, placebo-controlled, single-blind, and concealed allocation.

**Participants**

All COVID-19 patients admitted to Shahid Mohammadi Hospital in Bandar Abbas whose PCR test results are positive for SARS-Cov-2 and sign the written consent of the study are included in the study and immunocompromised patients, end-stage renal disease, moderate renal failure (clearance Creatinine 30 to 50 ml/min) or stage 4 severe chronic kidney disease or need for dialysis (creatinine clearance lesser than 30 ml/min), history of liver disease, hepatitis C infection or alcoholism, Glucose 6 phosphate dehydrogenase deficiency(G6PD), the ratio of Alanine transaminase to Aspartate transaminase 5 times above the normal limit, history or evidence of long QT segment on Electrocardiogram, psoriasis or porphyria, pregnancy, use of oral contraceptives, Dasatinib, Neratinib, Ozanimod, Pazopanib, Rilpivirine, Siponimod and/or Tizanidine and allergies to any study drug are excluded.

**Intervention and comparator**

Intervention group receives standard pharmacotherapy according to the treatment protocols of the National Committee of COVID-19 and oral famotidine 160 mg (Manufactured by Chemidarou Pharmaceutical Company) four times a day until the day of discharge, for a maximum of fourteen days. Comparator group receives standard drug therapy according to the treatment protocols of the National Committee of COVID-19 and placebo in the same dosage.

**Main outcomes**

Patients’ temperature, respiration rate, oxygen saturation, lung infiltration, lactate dehydrogenase and complete blood count were measured at the baseline (before the intervention) and on day 14 after the intervention or on the discharge day.

**Randomisation**

the person who has no role in admitting patients and assigning patients to random codes preparing random sequences using online tools and by permuted block randomization method. eligibility criteria are monitored by the person responsible for admitting patients. Codes in a random sequence are assigned to patients by the treatment team without knowing that each code is in the intervention or comparator group. Patient codes are then matched to randomly generated sequence information for interventions.

**Blinding (masking)**

All participants are unaware of which group of this study they are in and after grouping patients in the groups, Patients receive Famotidine in the treatment group and receive a placebo in the control group. The lead researcher, care givers, data collectors, and outcome assessors are aware of the grouping of patients.

**Numbers to be randomised (sample size)**

As there is no prior work on this research question, so no assumptions for the sample size calculation could be made. A total of 20 patients participate in this study, which are randomly divided into two groups of 10 as intervention or control groups.

**Keywords**

COVID-19, Randomised controlled trial, protocol, Famotidine, Hospitalized, Efficacy

**Introduction:**

Managing COVID-19 patients caused by the SARS-CoV-2 virus has become one of the challenges of care and treatment systems today. (1)

Recently, much research has focused on the design of vaccines and drugs for these patients. (2)

Famotidine is a cheap and widely available drug, a class of histamine 2 receptor antagonists that is widely used for its antacid properties. (1)

The results of computer simulation studies also identified famotidine as a possible inhibitor of 3-chymotrypsin-like protease in the SARS-Cov-2 virus. (2)

Famotidine can also alter subsequent signal pathways by binding to the histamine 2 receptor, including regulation of antibody production by B cells, the release of cytokines by Th1, differentiation and division of T cells, mast cell degranulation, and cell responses. Follow the dendritic (3)

Histamine 2 receptor antagonists have also shown successful results against human immunodeficiency virus (HIV), papillomavirus, and hepatitis B viruses (4).

Given all the above, there is still no clinical trial to determine the effect of famotidine on the severity and mortality of COVID-19 patients (5,6).

Therefore, considering the possible effects of famotidine on the immune regulation of COVID-19 patients, the antiviral effects of histamine 2 receptor antagonists, and the lack of clinical trial studies, in this one-blind randomized clinical trial studied the effect of famotidine on patients' improvement, symptoms and We review some laboratory features of COVID-19 patients.

**Methode:**

This study is a phase 3 randomized clinical trial, with the grouping of two parallel arms on 20 patients and the use of placebo in the control group, single-blinded, and randomization concealment that has been registered with the code IRCT20200509047364N2 to the Iranian Clinical Trial Registration Center ( IRCT). This study has also been approved by the Research Ethics Committee of Hormozgan University of Medical Sciences with the code IR.HUMS.REC.1399.255.

Data collection and recruitment is done in Shahid Mohammadi Hospital in Bandar Abbas. (Email: [shmh@hums.ac.ir](mailto:shmh@hums.ac.ir) , Web page: <https://shmh.hums.ac.ir/> )

**Eligibility criteria:**

**Inclusion criteria:**All COVID-19 patients whose disease has been confirmed by the PCR test for SARS-Cov-2. Signing the written consent of the study participant.

**Exclusion criteria:** All Immunocompromised patients, End-stage renal disease Moderate renal insufficiency (creatinine clearance 30-50 mL/min), stage 4 severe chronic kidney disease requiring dialysis (i.e creatinine clearance <30 mL/min), patients with a history of hepatic disease, History of hepatitis C infection, history of alcoholism, G-6-PD (glucose-6-phosphate dehydrogenase deficiency) ALT/AST >5 times the upper limit of normal, History of or evidence of QT prolongation on ECG examination History of psoriasis History of porphyria Pregnancy Use of oral contraceptive pills (OCP) Concomitant use of Dasatinib Concomitant use of Neratinib Concomitant use of Ozanimod Concomitant use of Pazopanib Concomitant use of Rilpivirine Concomitant use of Siponimod Concomitant use of Tizanidine Allergy to any study medication were excluded.

**Randomization:**

Before assigning groups to individuals eligible to participate in the study, informed consent is completed for grouping individuals. the person who has no role in admitting patients and assigning patients to random codes preparing random sequences using online tools (https://www.sealedenvelope.com/) and by permuted block randomization method. Individualized random allocation is done in blocks with sizes 2 and 4, and without stratification. eligibility criteria are monitored by the person responsible for admitting patients. Codes in a random sequence are assigned to patients by the treatment team without knowing that each code is in the intervention or placebo group. Patient codes are then matched to randomly generated sequence information for interventions. (randomization concealment is done by the treatment team without informing the person responsible for admitting patients and the person who prepared the random sequence.)

**Blinding description:**

In this study, all participants are aware of participating in this study and enter the study with their consent. All participants are unaware of which group of this study they are in and after grouping patients in the groups, Patients receive Famotidine in the treatment group and receive a placebo in the control group. The lead researcher, health care personnel, data collection officials, and those who evaluate the outcome are aware of the grouping of patients. Those who prepare the draft of the article are unaware of the groupings if they do not cooperate in the above cases.

**Sample size:**

Due to the lack of previous studies, the sample size of 20 patients who were divided into two groups of ten for intervention and control was used.

**Outcomes and measurement:**

Data collection is done by the medical team of Shahid Mohammadi Hospital and patients' records.

**Primary outcomes:**

Respiratory rate and Oxygen saturation state measured by Pulse oximeter, Lung infiltration status measured by Chest X-ray, Lactate Dehydrogenase(LDH) levels, C-reactive protein(CRP) level's, Lymphocyte count, and Platelet count measured by Pathobiology laboratory.

All primary outcomes were assessed At the beginning of the study (before the intervention) and day 14 after the intervention or the day of the patient's discharge.

**Secondary outcomes:**

Patient temperature status measured by a digital thermometer, Length of hospitalization, and length of Intensive Care Unit admission Obtained from the recording of patient information.

All secondary outcomes were assessed At the beginning of the study (before the intervention) and day 14 after the intervention or the day of the patient's discharge.

**Intervention groups:**

**Intervention group:**

The treatment group receives standard pharmacotherapy according to the treatment protocols of the National Committee of COVID-19 and oral famotidine 160 mg (Manufactured by Chemidarou Pharmaceutical Company) four times a day until the day of discharge, for a maximum of fourteen days. Vital signs of patients are also checked at regular intervals and frequently.

Standard pharmacotherapy according to the treatment protocols of the National Committee of COVID-19 includes Hydroxychloroquine / Chloroquine Phosphate: Hydroxychloroquine sulfate tablets 200 mg or chloroquine phosphate tablets 250 mg (equivalent to 150 mg base dose) 2 tablets every 12 hours on the first day and then one tablet every 12 hours for at least 7 days and up to 14 days. One of the following medications at the discretion and diagnosis of the treating physician: kaletra tablets (Lopinavir / Ritonavir) 50/200 mg every 12 hours 2 pieces after meals for at least 7 days and a maximum of 14 days. Tablets (Atazanavir / Ritonavir) 300/100 One tablet daily with food or Atazanavir 400 mg daily for at least 7 days and up to 14 days.

**Control group:**

The placebo group receives standard drug therapy according to the treatment protocols of the National Committee COVID-19 and placebo in the form of oral tablets four times a day, daily until patients are discharged, for a maximum of fourteen days.

**Statistical analysis:**

IBM-SPSS version 22 software will be used for data analysis, independent t-test and Mann-Whitney will be used to compare the means of quantitative data.

Chi-square and Fisher's test were used to compare qualitative variables.

**Trial Status**

Version 3 of the protocol was approved by the Deputy of Research and Technology and the ethics committee of Hormozgan University of Medical Sciences on August 2, 2020, with the local code 990245, and the recruitment started on August 17, 2020. recruitment ended on August 31, 2020.
**Trial registration**

The protocol was registered before starting subject recruitment under the title: The effect of Famotidine on the improvement of patients with COVID-19, at Iranian Registry of clinical trials (<https://www.irct.ir>) on 17 Agust 2020.

**Declarations**

**Ethics approval and consent to participate**

the protocol was approved by the ethics committee of Hormozgan University of Medical Sciences on August 2, 2020, with the code IR.HUMS.REC.1399.255. (<https://ethics.research.ac.ir/ProposalView.php?id=143967>)

Authors certify that this trial has received ethical approval from the appropriate ethical committee as described above. Informed consent will be obtained in Persian language. where the harms and benefits of the oral Famotidine and the placebo were described.

**Consent for publication**

Not applicable.

**Availability of data and materials**

The authors have not yet decided on the sharing of information.

**Competing interests**

The authors declare that they have no competing interests.

**Funding**

This study is supported by the Deputy of Research and Technology of Hormozgan University of Medical Sciences. there was no influence regarding the study design, collection, analysis and interpretation by the funding body. (Email: [research@hums.ac.ir](mailto:research@hums.ac.ir) , Web page:
<https://resv.hums.ac.ir> )

**References:**

1. Malone RW, Tisdall P, Fremont-Smith P, Liu Y, Huang X-P, White KM, et al. COVID-19: Famotidine, Histamine, Mast Cells, and Mechanisms. 2020 Jun 22 [cited 2020 Aug 23]; Available from: https://doi.org/10.21203/rs.3.rs-30934/v2

2. Janowitz T, Gablenz E, Pattinson D, Wang TC, Conigliaro J, Tracey K, et al. Famotidine use and quantitative symptom tracking for COVID-19 in non-hospitalised patients: a case series. Gut [Internet]. 2020 Jun 4 [cited 2020 Aug 23];69(9):gutjnl-2020-321852. Available from: http://dx.doi.org/10.1136/gutjnl-2020-321852

3. Freedberg DE, Conigliaro J, Wang TC, Tracey KJ, Callahan M v., Abrams JA, et al. Famotidine Use is Associated with Improved Clinical Outcomes in Hospitalized COVID-19 Patients: A Propensity Score Matched Retrospective Cohort Study. Gastroenterology. 2020 May 22;

4. Wu C, Liu Y, Yang Y, Zhang P, Zhong W, Wang Y, et al. Analysis of therapeutic targets for SARS-CoV-2 and discovery of potential drugs by computational methods. Acta Pharmaceutica Sinica B. 2020 May 1;10(5):766–88.

5. sen Gupta PS, Biswal S, Singha D, Rana MK. Binding insight of clinically oriented drug famotidine with the identified potential target of SARS-CoV-2 [Internet]. Journal of Biomolecular Structure and Dynamics. Taylor and Francis Ltd.; 2020 [cited 2020 Aug 23]. Available from: https://www.tandfonline.com/doi/abs/10.1080/07391102.2020.1784795

6. Shaffer L. 15 drugs being tested to treat COVID-19 and how they would work. Nature Medicine. 2020 May 15;


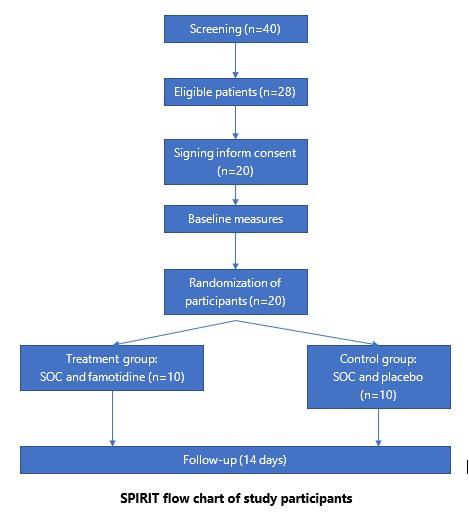

Supplement: Supplementary file 1 — Additional file 1. Full Study Protocol. [file 13063_2020_4773_MOESM1_ESM.docx]
